# Role of endogenous ACTH on circadian aldosterone rhythm in patients with primary aldosteronism

**DOI:** 10.1530/EC-14-0086

**Published:** 2014-09-19

**Authors:** Takuhiro Sonoyama, Masakatsu Sone, Naohisa Tamura, Kyoko Honda, Daisuke Taura, Katsutoshi Kojima, Yorihide Fukuda, Naotetsu Kanamoto, Masako Miura, Akihiro Yasoda, Hiroshi Arai, Hiroshi Itoh, Kazuwa Nakao

**Affiliations:** 1 Department of Medicine and Clinical Science, Kyoto University Graduate School of Medicine, 54 Shogoin Kawahara-cho, Sakyo-ku, Kyoto, 606-8507, Japan

**Keywords:** primary hyperaldosteronism, hypertension, adrenal gland, adrenocorticotropic hormone, clinical medicine

## Abstract

We recently reported that stimulation with high-dose ACTH caused different responses in terms of aldosterone secretion in aldosterone-producing adenomas (APAs) and idiopathic hyperaldosteronism (IHA) in patients with primary aldosteronism (PA). However, the role of endogenous ACTH in aldosterone secretion in PA has not been systematically evaluated. In this study, we examined diurnal changes in plasma aldosterone concentration (PAC), and changes in PAC after dexamethasone administration in patients with suspected PA, in order to evaluate the effect of endogenous ACTH on aldosterone secretion. Seventy-three patients admitted to Kyoto University Hospital with suspected PA were included. The patients were classified into non-PA, IHA, and APA groups according to the results of captopril challenge test and adrenal venous sampling. PAC at 0900 h (PAC_0900_), 2300 h (PAC_2300_), and after 1-mg dexamethasone suppression test (PAC_dex_) was measured and compared among the three groups. The PAC_2300_/PAC_0900_ and PAC_dex_/PAC_0900_ ratios were also analyzed. PAC_2300_ and PAC_dex_ were lower than PAC_0900_ in all three groups. There were no significant differences in PAC_2300_/PAC_0900_ among the three groups. However, PAC_dex_/PAC_0900_ was significantly lower in the APA group compared with the non-PA and IHA groups. The results of this study indicate that aldosterone secretion in APA patients is more strongly dependent on endogenous ACTH than in IHA and non-PA patients. The results also suggest that factors other than ACTH, such as clock genes, may cause diurnal changes in aldosterone secretion in IHA and non-PA patients.

## Introduction

Aldosterone secretion from the zona glomerulosa of the adrenal glands is controlled by several factors. Among these, angiotensin II (AII) and potassium are the two principal secretagogues for aldosterone (1). Both of these factors are known to stimulate aldosterone production by increasing the transcription of CYP11B2, the key steroidogenic enzyme of aldosterone synthesis. ACTH is also reported to stimulate aldosterone secretion, although its effects on aldosterone under normal conditions are mild compared with those of AII and potassium (1).

Primary aldosteronism (PA) is a major cause of secondary hypertension, affecting 5–10% of all hypertensive patients (2, 3, 4). In PA, aldosterone is autonomously secreted from the adrenal glands, and renin secretion from juxtaglomerular cells in the kidneys is suppressed (1). PA causes hypertension and sometimes hypokalemia as a result of increased aldosterone secretion (1), and PA is reportedly associated with increased rates of stroke, heart disease, and kidney failure (5, 6).

PA has two major subtypes, aldosterone-producing adenoma (APA), in which aldosterone hypersecretion occurs from a unilateral adrenal adenoma, and idiopathic hyperaldosteronism (IHA), in which aldosterone is secreted from bilateral adrenal hyperplasia of the zona glomerulosa (1). Although there are other rarer subtypes of PA, APA and IHA comprise more than 95% of all PA cases.

Aldosterone secretion in IHA cases is regulated by the renin–angiotensin system, as in normal and non-PA hypertensive cases (7). However, previous reports found that APAs were more responsive to ACTH stimulation than essential hypertension or IHA (8, 9, 10), even in AII-responsive APA cases (11). We recently reported that the ACTH stimulation test was useful for the diagnosis of APA among patients with essential hypertension and PA (12). The ACTH stimulation test involves evaluating the effects of 0.25 mg of 1–24 ACTH on plasma aldosterone concentrations (PACs). The ACTH test produces different aldosterone responses in patients with APA, IHA, and low-renin essential hypertension (12).

Previous reports documented a diurnal decline in PAC, and a decline in PAC following dexamethasone administration in PA and essential hypertensive patients (13, 14, 15), but these reports only analyzed a small number of cases and the selection of patients was not systematic, resulting in a risk of selection bias. Therefore, the role of endogenous ACTH on aldosterone secretion in patients with PA has not been clearly defined.

In the current study, we therefore examined the diurnal change in PAC, and the change in PAC after dexamethasone administration, in order to analyze the effect of endogenous ACTH on aldosterone in a series of patients with suspected PA.

## Subjects and methods

We retrospectively analyzed patients with suspected PA admitted to the Department of Endocrinology and Metabolism of Kyoto University Hospital, Kyoto, Japan, over an 8-year period since 2004. The study was approved by the Kyoto University Graduate School and Faculty of Medicine Ethics Committee and conducted in accordance with the principles of the Declaration of Helsinki. The patients with an aldosterone:renin ratio (ARR; ratio of PAC to plasma renin activity (PRA)) >555.6 pmol/l per ng/ml/h (i.e., 20 ng/dl per ng/ml/h) who were admitted to our hospital were initially included. All antihypertensive drugs except calcium channel blockers and α blockers were stopped at least 2 weeks before hospitalization (16). Also, mineralocorticoid receptor blockers were stopped at least 8 weeks before hospitalization (16). Patients with hypokalemia (i.e. serum potassium levels <3.5 mmol/l) were allowed to take oral potassium supplementation.

Blood pressure (BP) was measured in a quiet, warm room with patients being in the seated position with the arm held at heart level every morning and evening during hospitalization. The BPs given in Table 1 were obtained on the next morning and evening after hospitalization. All tests were performed during morning hours in a quiet room. PRA and PAC were measured in blood samples obtained in the morning after 30 min of rest in a supine position. The captopril challenge test was used to confirm the diagnosis of PA in this study. An ARR ≥555.6 pmol/l per ng/ml/h (i.e. 20 ng/dl per ng/ml/h) at 60 min after administration of 50 mg of captopril was considered positive for PA; a post-captopril ARR <555.6 pmol/l per ng/ml/h indicated non-PA (16).

The patients with confirmed PA underwent subtype diagnosis. A CT scanning of adrenal gland was performed for initial localization. Adrenal venous sampling (AVS) was used as the definitive test for subtype diagnosis. AVS was performed by expert radiologists using ACTH stimulation, as described previously (17). Adrenal vein cannulation was considered successful if the adrenal vein/inferior vena cava cortisol gradient (selectivity index) was >3.0. Lateralization was considered when the aldosterone:cortisol ratio (A/C) from one adrenal gland was at least three times greater than the ratio from the other adrenal gland (lateralization ratio, LR) and the A/C in the contralateral adrenal vein was lower than the A/C in the vena cava (contralateral ratio, CLR). Bilateral aldosterone secretion was considered when the CLR was >1.0 and the LR≤3.0 in a patient with confirmed PA. As shown below, patients with an ambiguous AVS outcome (i.e., LR>3.0 and CLR>1.0, or LR≤3.0 and CLR≤1.0) were excluded from this study.

APA diagnosis required that the following criteria were met: i) diagnosis of PA by captopril challenge test; ii) lateralization of aldosterone secretion at AVS; iii) CT evidence of adrenal mass and/or pathological evidence of adrenal adenoma in the adrenal gland with aldosterone hypersecretion; and iv) improvement of hypertension and hypokalemia after unilateral adrenalectomy or medical treatment with mineralocorticoid receptor blockers. Patients with confirmed PA for whom bilateral aldosterone hypersecretion was confirmed by AVS (i.e., LR≤3.0 and CLR>1.0) were diagnosed with IHA.

### Exclusion criteria

Patients with confirmed PA with no acceptable subtype diagnosis, unsuccessful AVS, ambiguous AVS outcomes, a negative post-captopril ARR with a pathologically confirmed APA, or autonomous cortisol secretion (i.e., plasma cortisol level ≥82.77 nmol/l after overnight 1-mg dexamethasone suppression test) were excluded from the study.

### Hormone measurement and dexamethasone suppression test

The levels of ACTH, cortisol, PRA, and PAC were measured in blood samples obtained after 30 min of rest in a supine position at 0900 (ACTH_0900_, F_0900_, PRA_0900_, and PAC_0900_ respectively) and at 2300 (ACTH_2300_, F_2300_, PRA_2300_, and PAC_2300_ respectively) on the same day. In addition, 1 mg dexamethasone was administered orally after blood sampling at 2300, and the levels of ACTH, cortisol, PRA, and PAC were measured after 30 min of rest at 0900 the following morning (ACTH_dex_, F_dex_, PRA_dex_, and PAC_dex_ respectively). The PAC_2300_/PAC_0900_ ratio and PAC_dex_/PAC_0900_ ratio were also analyzed.

For analysis, ACTH values <1.1 pmol/l were arbitrarily set to 1.1, and PRA values <0.1 ng/ml per h were set to 0.1.

### Statistical analyses

All the data were expressed as median (range). A Kruskal–Wallis one-way ANOVA followed by *post hoc* Steel–Dwass test was used to compare samples between groups. A *P* value <0.05 was considered to be statistically significant. The diagnostic accuracy of the 1-mg dexamethasone suppression test for APA was assessed by receiver-operating characteristics (ROC) curve and the area under the ROC curve (AUC). The optimal cut-off value (i.e., the best combination of sensitivity and lowest false-positive rate) was set at the closest point to the upper left corner of the ROC curve plot.

## Results

**Figure 1:**
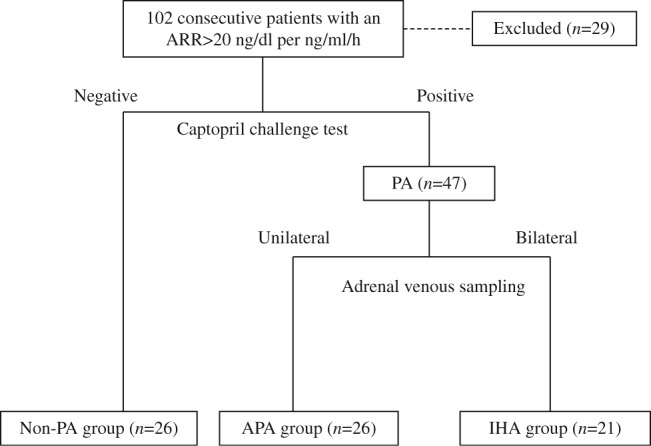
Flowchart of patient recruitment and the diagnosis of each group. ARR, aldosterone renin ratio; PA, primary aldosteronism; APA, aldosterone-producing adenoma; IHA, idiopathic hyperaldosteronism.

We analyzed 102 consecutive patients with positive screening tests for an ARR value >555.6 pmol/l per ng/ml/h who were admitted to our hospital. Of these, 26 patients were diagnosed with non-PA (non-PA group; i.e., screening-positive and confirmation-negative group) by captopril challenge test. The following patients were excluded from the study: six patients with confirmed PA who did not undergo AVS; nine patients with unsuccessful AVS; four with ambiguous AVS results; four with a negative post-captopril ARR with pathologically confirmed APA; and six patients with autonomous cortisol secretion. The remaining patients were classified as belonging to either the IHA or APA group by AVS (Fig. 1).

A total of 73 patients were included: 26 in the non-PA group, 21 in the IHA group, and 26 in APA group (Fig. 1). All but three of the APA patients underwent laparoscopic adrenalectomy, and all those who underwent surgery had pathologically confirmed adrenal adenomas.

### Baseline characteristics

The baseline characteristics of the IHA, APA, and non-PA groups are shown in Table 1. IHA patients tended to be older than APA or non-PA group. The patients with IHA and APA had significantly lower basal PRA levels than those of non-PA (*P*<0.05 and *P*<0.001 respectively). Basal PAC levels were significantly higher in the APA group compared with the IHA and non-PA groups (*P*<0.05 and *P*<0.005 respectively). The APA group also had significantly higher urinary aldosterone levels than the IHA and non-PA groups (*P*<0.01 and *P*<0.01, respectively). Serum K levels were significantly lower in the APA vs the non-PA group (*P*<0.001). 88.5% of the IHA group, 90.5% of the APA, and 80.8% of the non-PA group were taking antihypertensive agents. In the IHA group, 38.1% of patients were taking oral potassium supplementation; the figures in the APA and non-PA groups were 76.9 and 11.5% respectively. Morning and evening BPs were not significantly different among the three groups. Evening systolic BP (sBP) tended to be lower than morning sBP in APA group, while in IHA and non-PA group, evening sBP tended to be higher than morning sBP. The ratio of evening sBP to morning sBP in APA group was significantly lower compared with that in IHA group.

### Late-night hormone measurement and dexamethasone suppression test

**Figure 2:**
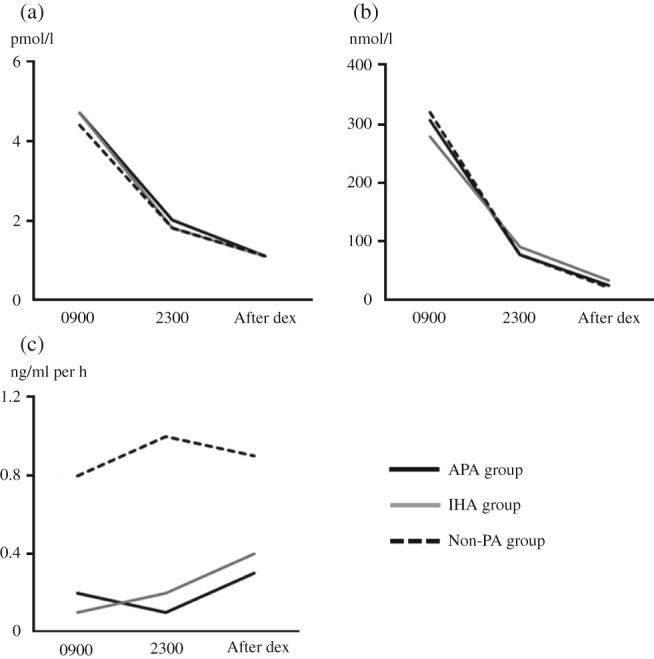
(a) Plasma ACTH level, (b) plasma cortisol level, and (c) plasma renin activity of non-PA group, IHA group, and APA group at 0900, 2300 and after dexamethasone administration. The median of each value is shown. ACTH, adrenocorticotropic hormone; F, cortisol; PRA, plasma renin activity; dex, dexamethasone.

The levels of ACTH, cortisol, PRA, and PAC at 0900 and at 2300 on the same day and at 0900 on the morning following dexamethasone administration are shown for the three groups (Fig. 2).

**Figure 3:**
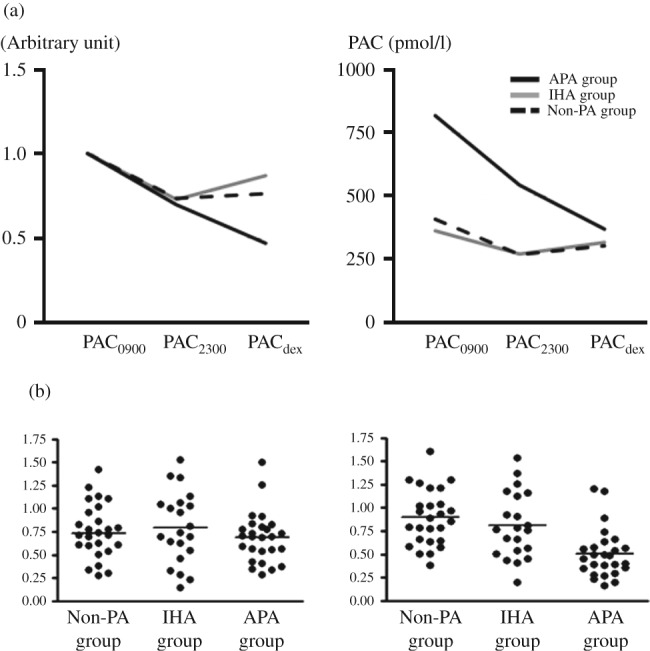
(a) PAC at 0900 and 2300, and PAC after 1 mg dexamethasone suppression test. Left panel: arbitrary units when PAC at 0900 was set to 1.0. Right panel: raw PAC values at each point. The median of each value is shown. (b) Scattergram of PAC_2300_/PAC_0900_ (left panel) and PAC_dex_/PAC_0900_(right panel) in non-PA, IHA, and APA groups. PAC, plasma aldosterone concentration; PAC_0900_, PAC at 0900; PAC_2300_, PAC at 2300; PAC_dex_, PAC at 0900 after 1 mg dexamethasone administration the previous night.

PAC_2300_ was significantly lower than PAC_0900_ in all three groups (Figs 2 and 3). PAC_2300_/PAC_0900_ was 0.72 (0.27–1.43) in the non-PA group, 0.73 (0.15–1.52) in the IHA group, and 0.70 (0.28–1.50) in the APA group. There were no significant differences in PAC_2300_/PAC_0900_ among the three groups. ACTH_2300_ was significantly lower than ACTH_0900_ in all three groups. Conversely, there were no significant differences between PRA_09__00_ and PRA_23__00_ in all three groups.

After dexamethasone suppression, PAC_dex_ was significantly lower than PAC_0900_ in all three groups. PAC_dex_/PAC_0900_ was 0.87 (0.37–1.61) in the non-PA group, 0.76 (0.19–1.54) in the IHA group, and 0.49 (0.16–1.20) in the APA group. In contrast to PAC_2300_/PAC_0900_, PAC_dex_/PAC_0900_ in the APA group was significantly lower than in the non-PA and IHA groups. ACTH_dex_ was suppressed to undetectable levels in most patients in all three groups. There were no significant differences between PRA_dex_ and PRA_0900_ among all three groups (Figs 2 and 3).

### Diagnostic accuracy of dexamethasone suppression test

As PAC_dex_/PAC_0900_ was significantly lower in the APA group compared with the non-PA and IHA groups, we analyzed the diagnostic accuracy of PAC_dex_/PAC_0900_ for the diagnosis of APA among the non-PA, IHA, and APA groups. Supplementary Figure 1(a), see section on supplementary data given at the end of this article shows the ROC curve for PAC_dex_/PAC_0900_ for the diagnosis of APA among the three groups. The AUC of the ROC curve was 0.821, with the optimal PAC_dex_/PAC_0900_ cut-off value of <0.58, corresponding to a sensitivity and specificity of 76.9 and 78.7% respectively (Supplementary Figure 1). When applying PAC_dex_/PAC_0900_ for the differential diagnosis between IHA and APA groups, the AUC of the ROC curve was 0.771, with the optimal PAC_dex_/PAC_0900_ cut-off value of <0.66, corresponding to a sensitivity and specificity of 66.7 and 80.8% respectively (Supplementary Figure 1(b)).

## Discussion

In this study, we examined the effect of endogenous ACTH on aldosterone secretion in consecutive patients with suspected PA by analyzing the diurnal change in PAC and the suppression of aldosterone after dexamethasone administration.

PAC was suppressed more strongly by 1 mg dexamethasone in the APA group than in the non-PA and IHA groups, indicating a greater dependency of aldosterone secretion on endogenous ACTH in APA patients compared with IHA and non-PA patients. These results are in accordance with previous studies (12, 13, 14, 15).

Meanwhile, analysis of late-night PAC revealed that all three groups underwent diurnal changes in aldosterone, and there were no significant differences among the three groups in terms of the reduction from PAC_0900_ to PAC_2300_. In addition, the diurnal changes in PAC in the non-PA and IHA groups were greater than the change in PAC after dexamethasone suppression test, though the diurnal change in ACTH was smaller than the change after dexamethasone administration. It is difficult to explain the diurnal change in PAC in these two groups solely in terms of the dependency of aldosterone secretion on endogenous ACTH, suggesting that other factors may be responsible for diurnal changes in PAC. One such candidate factor is renin, which is known to undergo diurnal changes (18). However, the patients analyzed in this study had low renin levels, especially in the case of the IHA group, and the effect of diurnal renin changes on aldosterone secretion is thought to be relatively small. The clock genes represent another candidate. A recent report by Doi *et al* (19). has shown that adrenal hyperplasia observed in IHA patients was immunoreactive for type I 3β-hydroxysteroid dehydrogenase (HSD3B1), whereas APA was immunoreactive for type II 3β-hydroxysteroid dehydrogenase (HSD3B2), not for HSD3B1. HSD3B2 is regulated by ACTH, but HSD3B1 is regulated by other factors, such as clock genes (20). We therefore hypothesized that aldosterone secretion from the zona glomerulosa in patients with IHA and low-renin essential hypertension is regulated not only by ACTH but also by clock genes. However, further studies are needed to address this hypothesis.

The diurnal change in PAC in the APA group was smaller than the change after dexamethasone suppression test, in accordance with the diurnal change in ACTH and the change after dexamethasone suppression test, suggesting that aldosterone secretion in APA patients is largely dependent on endogenous ACTH. This ACTH dependency supports our previous studies that the ACTH stimulation test is useful for detecting APA.

In prior years, the posture stimulation test was used to distinguish APA and IHA. According to Mulatero *et al*. (21), the sensitivity and specificity of the posture stimulation test for the diagnosis of APA between APA and IHA were 64 and 70% respectively , which was a little lower compared with dexamethasone suppression test in our study. The dexamethasone suppression is informative for the detection of APA, although it cannot be used for the final diagnosis of APA because of its relatively low diagnostic accuracy.

There were several IHA cases in which PAC declined to very low levels late at night or after dexamethasone suppression test, though we were unable to conclude if they were ACTH-responsive IHA (22) or bilateral APA cases because AVS cannot differentiate between IHA and bilateral APA cases. Also, there is a possibility that these cases included glucocorticoid remediable aldosteronism (GRA) cases (23). As we do not routinely check for GRA, we could not show the genetic background of all the PA patients in this study. One patient in the IHA group, who showed more than 80% fall of PAC after 1 mg dexamethasone administration, was tested for GRA, but was negative.

In conclusion, the results of this study indicate that aldosterone secretion in APA patients is more dependent on endogenous ACTH than in IHA or non-PA patients. Our results also suggest that factors other than ACTH cause diurnal changes in aldosterone secretion in IHA and non-PA patients.

## Supplementary data

This is linked to the online version of the paper at http://dx.doi.org/10.1530/EC-14-0086.

## Figures and Tables

**Table 1:** Baseline characteristics of the patients in each group.

**Parameter**	**IHA group**	**vs**	**APA group**	**vs**	**Non-PA group**
*n*	21		26		26
Age (years)	60 (36–69)	NS	46.5 (23–70)	NS	49.5 (34–70)
Sex (male:female)	9:12		15:11		15:11
Basal PAC (100.0–667.2 pmol/l)	416.7 (180.6–911.1)	*P*<0.05	805.6 (402.8–3833.3)	*P*<0.005	369.4 (227.8–925)
Basal PRA (0.2–2.7 ng/ml per h)	0.2 (0.1–1.5)	NS	0.1 (0.1–1.3)	*P*<0.001	0.65 (0.1–3.5)
U-Aldo (nmol/day) (<27.8 nmol/day)	26.7 (8.9–57.2)	*P*<0.01	43.3 (31.4–195.6)	*P*<0.01	32.1 (13.3–57.8)
Serum K (3.6–4.8 mmol/l)	3.6 (3.1–4.4)	NS	3.3 (2.3–4.3)	*P*<0.001	3.95 (3.1–4.3)
Morning systolic BP (mmHg)^a^	127 (98–165)	NS	132.5 (110–184)	*P*<0.05	122 (98–154)
Morning diastolic BP (mmHg)^a^	80 (54–102)	NS	85 (66–115)	NS	83.5 (61–113)
Evening systolic BP (mmHg)^a^	136 (107–161)	NS	127.5 (95–164)	NS	129 (102–161)
Evening diastolic BP (mmHg)^a^	80 (54–102)	NS	80 (66–115)	NS	82 (60–104)

Data are given as median (range). PAC, plasma aldosterone concentration; PRA, plasma renin activity; U-Aldo, urinary aldosterone; K, potassium; BP, blood pressure; NS, not significant.

a 80.8% of the non-PA group, 88.5% of the IHA group, and 90.5% of the APA group were taking calcium channel blockers and/or α blockers.
